# Molecule generation using transformers and policy gradient reinforcement learning

**DOI:** 10.1038/s41598-023-35648-w

**Published:** 2023-05-31

**Authors:** Eyal Mazuz, Guy Shtar, Bracha Shapira, Lior Rokach

**Affiliations:** Ben-Gurion University of the Negev, Beersheba, Israel

**Keywords:** Cheminformatics, Machine learning, Drug discovery and development

## Abstract

Generating novel valid molecules is often a difficult task, because the vast chemical space relies on the intuition of experienced chemists. In recent years, deep learning models have helped accelerate this process. These advanced models can also help identify suitable molecules for disease treatment. In this paper, we propose Taiga, a transformer-based architecture for the generation of molecules with desired properties. Using a two-stage approach, we first treat the problem as a language modeling task of predicting the next token, using SMILES strings. Then, we use reinforcement learning to optimize molecular properties such as QED. This approach allows our model to learn the underlying rules of chemistry and more easily optimize for molecules with desired properties. Our evaluation of Taiga, which was performed with multiple datasets and tasks, shows that Taiga is comparable to, or even outperforms, state-of-the-art baselines for molecule optimization, with improvements in the QED ranging from 2 to over 20 percent. The improvement was demonstrated both on datasets containing lead molecules and random molecules. We also show that with its two stages, Taiga is capable of generating molecules with higher biological property scores than the same model without reinforcement learning.

## Introduction

A major challenge in drug discovery is designing drugs with the desired properties. The chemical space of potential drug-like molecules is between $10^{23}$ to $10^{60}$, of which about $10^8$ of molecules are synthesized^1,2^. Additionally, the average cost of developing a new drug is one to two billion US dollars, and the average development time is 13 years^3^. Traditionally, chemists and pharmacologists use their intuition and expertise to identify new molecules^4^. While Lipinski’s “rule of five”^5^ may reduce the number of possible drug-like molecules, the search space remains large. In order to narrow the space further, high-throughput screening (HTS) is used; however, the task remains daunting. Additionally, computational methods can be used to narrow the drug search space and shorten the time needed to develop new drugs.

In recent years, there have been many attempts to use deep learning, particularly generative models, for drug design^6,7^. However, the task of generating optimized and valid molecules using computational methods remains challenging due to the large search space and the small number of labeled samples.

There have been several attempts to use SMILES (simplified molecular-input line-entry system) strings as a representation for molecules^8^. For example, Gomez et al. tried using generative models based on SMILES strings for the molecule generation task. However, the proposed methods only managed to generate a low percentage of molecules that were considered valid due to the complicated grammatical rules of SMILES^6,9^.

Recently, the use of reinforcement learning (RL) has gained attention due to its ability to solve a wide range of problems such as playing the game of Go^10^ and operating machines. RL systems excel in these tasks thanks to their ability to make sequential decisions and maximize defined long-term rewards; this allows for the direct optimization of desirable new drug properties that are not derived from the model itself when using generative models such as recurrent neural networks (RNNs).

Later, RL optimization was incorporated into SMILES generation methods to generate molecules with desired properties, such as high IC50 values for JAK2, using a recurrent neural network (RNN)^11^ such optimization is technically challenging, since it tends to cause the model to converge toward a set of primarily invalid molecules, since RNNs cannot handle long sequences.

To improve the rate of valid molecules generated, other studies constrained the input of generative models when producing molecules by forcing the model to adhere to certain rules when generating molecules. Some studies proposed the use of variational autoencoders (VAEs) to generate valid molecules by learning the distribution of a latent space and sampling from it, instead of sequentially generating the molecule token by token^12,13^. However, the validity rate of these methods was relatively low; these results could be explained by the lower validity rate obtained in those studies for unseen molecules compared to known ones. To address this issue, the authors proposing the junction tree variational autoencoder (JTVAE)^7^ represented molecules as junction trees in order to encode the sub-spaces of the molecular graph. This allowed the decoder to generate valid molecules by utilizing only valid components while considering how they interact.

Two approaches exist to deal with the issue of long sequences presented by RNN models. The first is the use of factional-based architectures which are a wavelet-based architecture that compresses the associated operator’s kernel using fine-grained wavelets and thus handles long sequence data^14,15^. The other approach, which is now de-facto the gold standard in neural language processing (NLP), is the use of transformer models^16,17^ which achieve state-of-the-art results on many NLP tasks.

In this paper, we propose a new RL-based method to generate molecules with desired properties, which overcomes the problem of generating valid molecules with desired properties. We use a transformer-based architecture, utilizing SMILES string representations in a two-stage approach. To the best of our knowledge, this is the first application that utilizes both transformer models and reinforcement learning together for molecule graph generation. In the first stage, the model learns to embed these discrete string representations in a vector space. Then, in the second stage, the model optimizes the vector space in order to generate molecules with the desired properties, such as QED (quantitative estimate of drug-likeness) or pIC50. The use of an attention mechanism allows our method to gain an understanding of the underlying chemical rules that make a valid molecule by performing a simple language modeling task, using just a small amount of data. Then, the understanding gained regarding those rules, along with policy gradient RL, is used to generate molecules with the desired properties.

We evaluate our model on multiple datasets with various properties on the tasks of molecule generation and optimization for the desired properties and compare it to several state-of-the-art approaches^1–3,7,18,19^ that use different representations and techniques for molecule generation. We demonstrate our model’s ability to generate a high percentage of valid molecules and rival methods that use other techniques to ensure the generation of valid molecules. Additionally, unlike previous research that only focuses on the top molecules generated, we show our model’s ability to generate large number of molecules with a high mean QED, which defines how drug-like a molecule is, while maintaining a low synthetic accessibility score, a theoretic score of how hard it is to synthesize the molecule.

**Figure 1:**
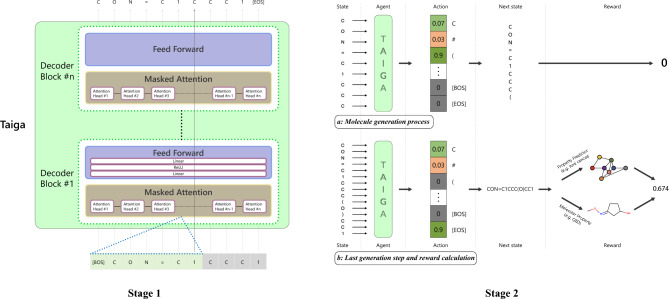
Overview of the training process. Stage 1: We train the transformer model on the language-modeling task of predicting the next token , e.g. in the figure when the last input token is ‘1’ Taiga tries to predict the next token in the sequence which is ‘C’, since it’s an auto-regressive model, it can only attend to previous tokens, i.e. those in the green squares indicated by the blue dotted line, and not the subsequent tokens in the gray squares. The next token prediction is done in parallel because of the attention mechanism. Stage 2: (**a**) The agent receives the SMILES string and predicts the next token by sampling from the output distribution, which is then appended to the SMILES string to create the next state. A reward of zero is given to the molecule at any step that is not the final step. (**b**) The agent completes the molecule generation process by predicting a [EOS] token (which is not appended to the SMILES string), and a reward greater than zero is provided either by computing the property directly from the molecule by using libraries such as RDKit or by feeding the generated molecule to an external property predictor (e.g. Chemprop) which generates the reward for the molecule.

In the task of optimizing a biological property (i.e., IC50), we show that Taiga is capable of improving existing molecules and generating molecules with the desired biological properties. Our main contributions are as follows:

- Introducing Taiga, an RL-based method that utilizes the transformer architecture, which is capable of generating novel and diverse molecules.
- Demonstrating that the use of an attention mechanism combined with policy gradient RL can overcome the existing challenges of generating valid molecules represented as SMILES strings.
- Performing extensive experiments using several datasets with a range of properties and multiple metrics to evaluate the performance of our method’s components (Fig. 1).

**Table 1:** Overall training dataset statistics.

Dataset	Number of molecules	QED	SAS	Max atoms
GDB13 rand	1,000,000	0.513	4.96	13
Moses	1,584,663	0.810	2.45	30
Zinc	249,456	0.731	3.05	38

## Results

In this section, to evaluate Taiga’s performance, we validate it on 2 common tasks: molecule generation and molecule optimization, for the molecule generation task we compare Taiga to the performance of several SOTA baselines using multiple datasets and metrics.

**Table 2:** Performance comparison on the property generation task; the mean score and standard deviation of the molecules is presented.

Dataset	Model	QED($\uparrow$)	SAS($\downarrow$)	Validity($\uparrow$)	Diversity($\uparrow$)	Novelty($\uparrow$)
GDB13	GCPN${*}$	–	–	–	–	–
	JTVAE	0.49±0.10	4.88±0.64	**100%**	89%	**100%**
	MolGPT	0.50±0.12	4.93±0.85	90%	**100%**	99%
	MolGAN	0.50±0.11	4.98±1.08	89%	8%	99%
	GraphDF	0.38±0.13	4.93±0.83	**100%**	99%	99%
	LSTM	0.51±0.12	**3.88**±0.83	99%	96%	96%
	Taiga	**0.64**±0.11	4.87±0.78	95%	**100%**	**100%**
Moses	GCPN	0.64±0.15	4.38±0.85	99%	99%	99%
	JTVAE	0.70±0.12	4.24±0.70	**100%**	**100%**	**100%**
	MolGPT	0.75±0.11	2.88±0.58	62%	98%	**99%**
	MolGAN$${**}$$	–	–	–	–	–
	GraphDF	0.42±0.13	4.76±0.80	**100%**	99%	99%
	LSTM	0.80±0.07	**2.06**±0.46	95%	90%	82%
	Taiga	**0.83**±0.07	2.25±0.49	97%	99%	95%
Zinc	GCPN	0.65±0.15	4.53±0.86	99%	99%	99%
	JTVAE	0.64±0.14	4.69±0.76	**100%**	**100%**	**100%**
	MolGPT	0.67±0.16	3.98±0.99	57%	99%	99%
	MolGAN	0.33$\pm 0.00$	5.23$\pm 0.00$	**100%**	0%	**100%**
	GraphDF	0.42±0.13	4.80±0.91	99%	99%	**100%**
	LSTM	0.73±0.14	**2.47**±0.88	56%	96%	96%
	Taiga	**0.75**±0.11	2.89±0.92	88%	98%	97%
–	MolDQN$${***}$$	0.78±0.10	6.31±0.83	**100%**	65%	–

Significant values are in bold.

$*$The authors support the GDB dataset in their code, however we were unable to run the code; our attempt to contact the authors was unsuccessful.

$$**$$It is infeasible to create training data from the Moses dataset, even when using more than 128GB of RAM.

$$***$$MolDQN is uses only RL and does not need a training set; therefore, it does not have a novelty score and is listed separately.

### Molecule generation

*1. Data:* We used three datasets in our experiments: the Moses^20^, Zinc^21^, and GDB13^22^ datasets.

- The **Moses** dataset consists of 1.6M training set molecules extracted from the Zinc clean lead collection.
- The **Zinc** dataset consists of 250K molecules extracted from the Zinc database.
- The **GDB13 rand** dataset consists of 1M random molecules extracted from the larger GDB13 database.

These datasets differ from one another in terms of the number and type of molecules included. Experimenting on these different datasets allows us to demonstrate the generalization ability of the methods evaluated.

*2. Baselines:* We compared our method to various approaches: (1) GCPN^1^, a method that uses proximal policy optimization (PPO) on molecular graphs, with the addition of adversarial loss; (2) JTVAE^7^, a method which uses the junction tree algorithm to generate molecular graphs with autoencoders; (3) MolGPT^2^, a method that generates SMILES using transformer architecture only; (4) MolGAN^18^, a method that generates molecular graphs using GANs and DDPG; (5) MolDQN^3^, a method that works on molecular graphs using Q-learning; (6) GraphDF^19^ a discrete normalizing flow method; and (7) an LSTM version of our method which also uses policy gradient RL, similar to^11^. We ran all models with their released code, optimizing for the same target property (i.e., QED), each with its respective reward function, as described in the original papers, on the same hardware containing one TITAN RTX GPU, 60GB RAM, and an eight-core CPU.

*3. Model Configuration:* Taiga consists of four decoder layers, each with eight attention heads, a feedforward size of 1024, and an initial embedding size of 512, as those parameters are frequently used in transformer models and were not optimized during this research. The model was trained for three epochs on the language modeling task and 100 steps, with each step averaging 500 episodes (molecules) in the RL stage. We use the REINFORCE algorithm with a discount factor of 0.99 and a maximum sequence size of 150 characters, we used character-level tokenization for all our experiments. The LSTM-PG uses the same hyperparameters as Taiga for all experiments.

We designed the following reward function for our model:

1 $$\begin{aligned} R(s_T)= {\left\{ \begin{array}{ll} 10 * QED(s_T), &{} \text {if}\ s_T \ \text {is valid molecule} \\ 0, &{} \text {otherwise} \end{array}\right. } \end{aligned}$$

*4. Metrics: * We utilized the following widely used metrics to evaluate the methods:

- *Validity*: the percentage of valid molecules a model is able to generate. If RDKit was able to process the SMILES string and generate a Molecule object, then the validity of that molecule is set to 1. Otherwise, validity is set to 0.
- *Novelty*: the percentage of molecules generated that do not appear in the training data.
- *Diversity*: the percentage of unique molecules the model can generate .
- *Quantitative Estimation of Drug-Likeness* (QED), the geometric mean of eight common molecule properties (molecular weight, AlogP, hydrogen bond donors, hydrogen donor acceptors, molecular polar surface area, rotatable bonds, aromatic rings, structural alerts), that can estimate how well the molecule behaves in the human body, This value can be range from 0 to 1, and the higher the QED value, the similar a molecule is to existing drugs and will have higher chance surviving the drug-discovery process^23^.
- *Synthetic Accessibility Score* (SAS), an estimation of the how easy it is to synthesize a molecule by calculating the fragment score penalized by the complexity of the molecule; it ranges from 0 to 10, where a molecule with an SAS value above 6 is considered challenging to synthesize^24^.

We calculated the QED and SAS metrics after removing all of the invalid molecules from the set of generated molecules. Novelty, Validity and Diversity are all crucial metrics for assessing performance of generative models in the drug discovery. These metrics have different use meanings, since a model can have 100% diversity with 0% novelty by just generating all molecules from the dataset it was training on. and vice versa, a model can have 100% novelty but 0% diversity by generating only 1 unique molecule but generating over and over again. For all methods, we generated the molecules after the optimization stage. For each method, we generated 25K molecules to calculate the metrics.

**Figure 2:**
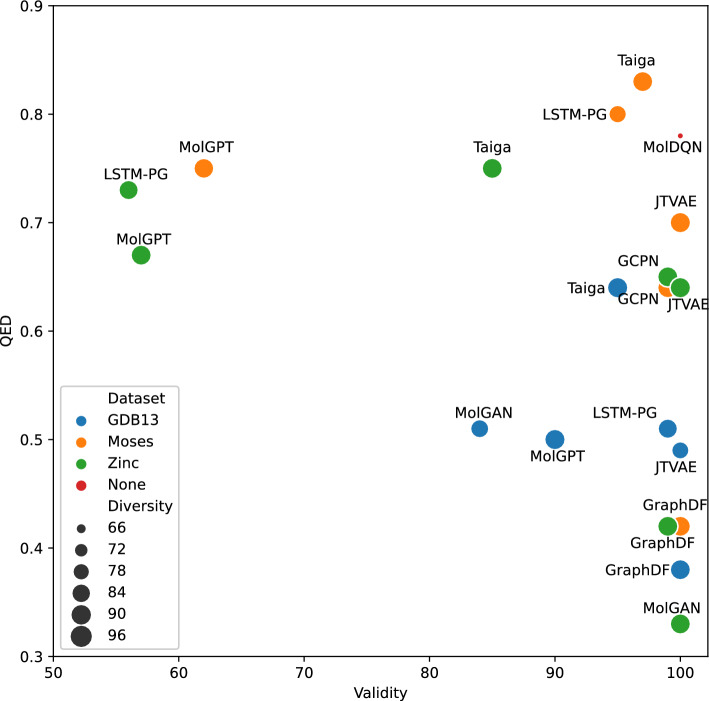
Models’ QED performance as a function of the molecule validity rate. The size of the dot represents the diversity value (higher is better); models that are closer to the top-right corner are considered better.

*5. Results: * Table 2 , Figure 2 and Supplementary Figure S1 presents the results for molecule generation and optimization across all models and datasets , and the aggregate results of the validity, QED, and diversity for all models. Supplementary Table S1 and Supplementary Figure S2 present the results of the top-3 molecules generated with each dataset in terms of their respective QED scores. As can be seen in Table 2 and Supplementary Figure S1, on the GDB13 dataset, which has a lower mean QED than the other datasets (see Table 1), Taiga is the only method that was able to obtain QED scores higher than the dataset’s mean QED score. However, Taiga wasn’t able to improve the SAS scores of the generated molecules relatively to the dataset’s mean SAS score, and achieved lower SAS scores than LSTM-PG which achieved the best SAS scores for this dataset. This is probably Due to the fact that the GDB13 1m rand dataset is a random subset of the entire GDB13 dataset and is not preprocessed to contain lead-like molecules, thus making it more challenging for optimization and reaching good performance. On this dataset, Taiga also achieved the best scores in terms of novelty and diversity while maintaining a high validity score, this means that the molecules generated are not only valid but high novelty score means we have higher chance to come across a lead molecule (since high novelty mean it doesn’t overfit and generates molecules from the training set). As seen in Supplementary Table S1 and Supplementary Figure S2 our method excels and is the only one that generated molecules with a QED value above 0.9. When looking at the SAS scores of the best molecules in terms of their QED in the GDB dataset, we find mixed results; this most likely occurred, because none of the methods directly tries to optimize the SAS score, and therefore when a model generates molecules, it generates compounds that are more complex and thus have a higher QED score but are harder to synthesize.

Of the three examined datasets, Taiga achieved the best optimization results with the Moses dataset, while still maintaining a high value for validity, diversity, and novelty metrics averaging around 97%. Compared to graph-based methods like JTVAE and GCPN, which represent molecules as complex graphs to generate a high rate of valid molecules, our method achieved comparable results on the diversity and novelty metrics and was not far behind on the validity metric meaning that SMILES-based method can generate high percentage of valid molecules. On the majority of metrics, our method performed the same or better than SMILES-based methods such as MolGPT and LSTM-PG, but MolGPT was able to achieve higher novelty score for the Zinc and Moses datasets. In Supplementary Table S1, we can see that Taiga generated molecules with a higher QED value than that obtained by graph-based methods and other SMILES-based methods, while LSTM-PG did manage to created a high amount of valid molecules and achieved the best SAS scores on the Moses dataset (due to the fact it generated molecules which lower QED score which are less complex), it wasn’t able to improve the metric it was optimized for which means that it can’t generate both optimized and valid molecules which further emphasizes the limitation of LSTM-based method in comparison to Transformers.

On the Zinc dataset (see Table 2 and Supplementary Figure S1), most methods generated molecules with an average QED value similar to the dataset mean and a high SAS score, but Taiga generated molecules with a QED value higher than the mean. Although graph-based methods such as GCPN and JTVAE were able to achieve a higher value on the validity metric than SMILES-based methods with this dataset, however, they achieved lower mean QED scores. This makes them less favorable for generating potential molecules even if they obtained a perfect validity. In addition, we can see that Taiga’s validity scores are higher than those of MolGPT and LSTM-PG which strengthen the idea of using transformers and RL together in a 2-stage training scheme. Although MolGAN achieved numerical results on this dataset, the SMILES strings it generated was only “.*.*.*.*.*.*.*” which was accepted as valid by RDKit but is not practical in any sense.

In Supplementary Table S1S1 and Supplementary Figure S2, we can see that Taiga obtained the best QED scores on the top molecules, while other methods such as LSTM-PG, GCPN, and MolGAN failed to obtain good QED scores for top molecules. In addition, in terms of the SAS score, Taiga demonstrated superiority over almost all other methods, obtaining better SAS scores for most molecules aside from LSTM-PG, which was able to generate molecules with better SAS scores. This is due to the fact that LSTM-PG can’t handle long sequences and generate shorter SMILES which result in smaller molecules with less atom and bonds which have a lower complex structure and thus easier to synthesize, since the SAS score penalize for complexity.

**Table 3:** Performance on the property optimization task. Baseline refers to the results obtained after the language modeling stage and before RL. Maximized refers to the results obtained after the language modeling stage and RL stage calculated by the raw output of the property predictor.

Dataset	Type	Metric	Value($\uparrow$)	QED($\uparrow$)	SAS($\downarrow$)	Validity($\uparrow$)	Diversity($\uparrow$)	Novelty($\uparrow$)
GDB13	Baseline	pIC50	5.180	0.521	5.07	97.49%	99.99%	99.91%
	Maximized	pIC50	**10.462**	0.563	5.03	97.84%	86.58%	86.58%
	Baseline	Anti-cancer	0.158	0.521	5.07	97.49%	100.00%	99.89%
	Maximized	Anti-cancer	**0.844**	0.552	4.65	95.54%	99.99%	99.99%
Moses	Baseline	pIC50	4.949	0.805	2.42	93.72%	99.87%	89.55%
	Maximized	pIC50	**5.612**	0.795	2.41	95.52%	99.78%	95.59%
	Baseline	Anti-cancer	0.304	0.805	2.42	93.77%	99.87%	89.55%
	Maximized	Anti-cancer	**0.774**	0.749	2.12	96.43%	98.12%	93.20%
Zinc	Baseline	pIC50	4.936	0.727	3.09	74.94%	99.98%	99.93%
	Maximized	pIC50	**6.333**	0.671	2.99	92.33%	93.32%	93.22%
	Baseline	Anti-cancer	0.215	0.726	3.09	74.94%	99.99%	99.99%
	Maximized	Anti-cancer	**0.824**	0.782	1.98	93.35%	87.84%	87.96%

Significant values are in bold.

Compared to MolDQN, which achieved better QED scores than Taiga on two of the three datasets without using a dataset during the training process, MolDQN achieved the lowest diversity scores out of all methods, which means that MolDQN is unable to generate a diverse set of molecules and generates the same molecule repeatedly. Similarly, it achieved the lowest SAS score out of all methods, thus generating molecules that are difficult to synthesize. This is due to the fact that MolDQN is a Q-learning algorithm, which at test time uses a greedy approach, and chooses actions based on the highest Q-values when generating molecules.

Aggregating the results, as seen in Fig. 2, shows Taiga’s superior performance across all datasets. Taiga is located in the upper-right corner of the figure (high validity, high QED) and has high diversity (indicated by a larger circle). Most of the examined methods were unable to generate a large amount of valid molecules with high QED values; some methods (e.g., JTVAE) were able to achieve good validity and diversity scores but at the cost of degraded performance on the target properties that the model tried to optimize. On the other hand, Taiga achieved better performance on the target task of optimizing for the desired property at the cost of a slightly lower validity rate.

### Property optimization

In this subsection, we evaluate Taiga’s ability to optimize biological properties with therapeutic function, which are harder to predict than QED; such a task requires additional supervised learners to predict molecular properties.

*1. Data:* We used 2 datasets, the first is IC50 data extracted from the ChEMBL database^25^ and extracted all of the molecules that have exact pIC50 values, i.e., we removed molecules for which only a range is available. pIC50 is the negative log of the IC50 value by using the following formula: $9- log_{10}(IC50)$. We focused specifically on the BACE (Beta-secretase 1) protein. After filtering out 10,164 molecules, we ended up with 9,331 samples with exact pIC50 values. The second is a dataset of molecules that used in cancer treatments, we collected around 400 molecules from various sources that had indication for some anti-cancer activity (FDA approval, clinical trials, DrugBank, etc. ), and were assessed as such by a pharmacologist and around 1000 molecules that are not known for treating cancer were randomly sampled from the list of FDA approved drugs after filtering after filtering out drugs that were already examined in cancer-related clinical trials (as reported in ClinicalTrials.gov) or drugs that are chemically similar to those drugs.

*2. Model Configuration:* We used the same configuration for Taiga as the one for the molecule generation task. For the property prediction model we utilize Chemprop^26^, a message passing graph neural network (MPNN) since its ability to predict potential molecules^27^. We train the model with the default parameters the library (https://github.com/chemprop/chemprop/.) provides, that is for 30 epochs with a batch size of 50, learning rate of 1e−4 and ensemble size of 2, using supervised learning on the curated dataset.

Inspired by Popova et al.^11^, we used the following reward function for the IC50 task:

2 $$\begin{aligned} R(s_T)= {\left\{ \begin{array}{ll} exp\left( \frac{pIC50}{3}\right) , &{} \text {if}\ s_T \ \text {is valid molecule} \\ 0, &{} \text {otherwise} \end{array}\right. } \end{aligned}$$

and for the anti-cancer prediction we use the following reward function:

3 $$\begin{aligned} R(s_T)= {\left\{ \begin{array}{ll} Chemprop(s_T), &{} \text {if}\ s_T \ \text {is valid molecule} \\ 0, &{} \text {otherwise} \end{array}\right. } \end{aligned}$$

where $Chemprop(s_T)$ is the raw output probability of the MPNN which ranges between 0 and 1 model raw output probabilities.

*3. Results:* The results presented in Table 3 demonstrate Taiga’s ability to maximize pIC50 values with the different datasets. We can see that when using all of the datasets as baselines, Taiga can be optimized for biological properties. On average, Taiga increased the pIC50 value by 20% when converting to IC50 values; this is the equivalent of reducing the concentration by a factor of 3-5 for the same therapeutic effect. We can see that the validity constraint of equation 2 helps maintain the same validity scores as the baseline. This prevents overfitting by generating random strings that can exploit the property predictor. Looking at other metrics such as the QED or SAS, we can see that Taiga was able to generate molecules with improved pIC50 values and at the same maintained similar SAS and QED values to those of the baseline; this means that it did not only do the molecules have a better potential for treatment, they are also easy to synthesize and have drug-like properties. With two of the three datasets, Taiga was able to keep generating a set of novel and diverse molecules after the RL stage, and on the Zinc and GDB dataset, the novelty and diversity scores decreased but by just a small margin. This means that Taiga was able to generate a set of molecules with higher pIC50 values while ensuring that the molecules are different from each other and were not seen during the training process.

When generating molecules to have anti-cancer activity, we also see that Taiga can maximize and generate molecules with high potential for cancer treatments without compromising other metrics, When calculating molecular similarity to existing anti-cancer therapeutics, the top molecules generated are chemically similar, which means that Taiga did manage to learn some understanding on what makes a drug anti-cancer. We also see Taiga’s manages to generate a high amount of novel and diverse molecules while maintaining a high validity rate. Looking at the other metrics, such as QED or SAS, we can see that Taiga can generate anti-cancer molecules while maintaining around the same SAS score but having lower QED scores. When looking at anti-cancer molecules, some of them violate Lipinski’s rule of 5, so it makes sense to have lower QED (which as part of its average uses properties from the rule of 5) scores as a trade-off for anti-cancer activity.

**Table 4:** Comparison of the results for the main metrics for Taiga, with and without the RL stage.

	GDB13			
	Validity($\uparrow$)	Divesity($\uparrow$)	Novelty($\uparrow$)	QED($\uparrow$)
w/o RL	97.78%	99.99%	99.90%	0.510
w/ RL	95.82%	100.00%	100.00%	0.646

Stage	Moses			
	Validity($\uparrow$)	Divesity($\uparrow$)	Novelty($\uparrow$)	QED($\uparrow$)
w/o RL	92.82%	99.40%	94.17%	0.806
w/ RL	97.21%	98.90%	95.56%	0.831

	Zinc			
	Validity($\uparrow$)	Divesity($\uparrow$)	Novelty($\uparrow$)	QED($\uparrow$)
w/o RL	69.13%	99.97%	99.94%	0.733
w/ RL	85.46%	99.70%	99.60%	0.757

**Table 5:** Comparison of the results for the main metrics for Taiga, with different tokenizer schemes.

Tokenizer	GDB13				
	Validity($\uparrow$)	Divesity($\uparrow$)	Novelty($\uparrow$)	QED($\uparrow$)	SAS($\downarrow$)
BPE	94.48%	100.00%	100.00%	0.625	5.38
Char	95.82%	100.00%	100.00%	**0.646**	**4.87**

	Moses				
	Validity($\uparrow$)	Divesity($\uparrow$)	Novelty($\uparrow$)	QED($\uparrow$)	SAS($\downarrow$)
BPE	97.38%	99.86%	86.78%	0.816	2.44
Char	97.21%	98.90%	95.56%	** 0.831**	**2.25**

	Zinc				
	Validity($\uparrow$)	Divesity($\uparrow$)	Novelty($\uparrow$)	QED($\uparrow$)	SAS($\downarrow$)
BPE	96.13%	99.40%	99.21%	**0.794**	**2.58**
Char	85.46%	99.70%	95.60%	0.757	2.89

Significant values are in bold.

### Ablation study

We conducted an ablation study to evaluate the contribution of the RL stage on Taiga’s performance. As can be seen in the results presented in Table 4, with the Moses dataset, before the RL stage Taiga underperformed in terms of validity and novelty when generating molecules; in addition, the QED value obtained was similar to the mean QED of the dataset (see Table 1). However, after the RL stage, the model was able to find a policy that enables better maximization of the QED. This is demonstrated by the increase in the mean QED of the molecules generated and the increase in the validity and novelty scores.

**Table 6:** Cross-dataset novelty scores of the molecules generated. Rows indicate the source of the pre-training dataset used to train Taiga and generate molecules with. Columns indicate the actual dataset we compare novelty scores with.

		Test dataset		
		Moses	Zinc	GDB13
Train dataset	Moses	95.56	98.74	98.90
	Zinc	98.82	95.60	99.70
	GDB13	100.00	100.00	100.00

We can see a similar phenomenon with the GDB dataset when comparing the results before and after the RL stage. Although the model was able to generate more valid molecules before the RL stage, the difference in the mean QED value obtained before and after the RL stage emphasizes the fact that the model was able to learn how to generate highly optimized molecules with just a slight trade-off in terms of the validity.

A similar improvement was seen with the Zinc dataset; before the RL stage, Taiga struggled to generate valid molecules and obtained a mean QED similar to that of the dataset itself. After the RL stage, Taiga generated more than 15% more valid molecules without significantly compromising the performance in terms of the diversity and novelty metrics; its mean QED also improved.

Another setting we tested is using RL directly without incorporating the language modeling task first. The results are not included in the table since the model wasn’t able to converge at all. After the first 20 steps it reached a point where the it failed to generate valid molecules (or any molecular formula at all).

We also tested the effects of using different tokenizers on Taiga’s ability to generate optimized molecules. We switched the character-level tokenizer for a BPE tokenizer^28^. In the end, the vocabulary consisted of 500 tokens (excluding special tokens such as [BOS], [EOS], etc.).

Table 5 summarize the results of our experiments. As can be seen, using the BPE tokenizer resulted in lower QED and SAS scores for both the GDB and Moses datasets. On the Zinc dataset, we can see that both QED and SAS scores have been improved by the BPE tokenizer. Moreover, it improved the model’s ability to generate valid molecules.

I believe this is due to the fact that the zinc dataset contains the greatest number and diversity of molecules. It can be determined by examining the number of unique single characters in each dataset (Zinc: 34, Moses: 26, GDB: 21) that are used as the initial vocabulary when training the BPE tokenizer. If the initial vocabulary and dataset are not diverse enough, the vocabulary will end up with similar tokens, but longer (e.g. CCCC and CCCCCC). As a result, the model learns very short sequences, however on the Zinc dataset, because of its large initial vocabulary and diverse set of molecules, we will still be able to learn longer sequences with a large vocabulary that is sufficiently diverse.

This improves the validity score since the sequences are shorter than the character-level and each token contains a group of atoms (and bonds), so the model has lower chances of generating invalid molecules compared to using character-level tokens, where each token can cause the molecule to become invalid.

To further assess Taiga’s ability to generate novel molecules and not overfit to the training data, for each dataset we calculated the novelty scores of the molecules generated during the molecule generation stage based on the other datasets. As seen in Table 6, Taiga generated novel and unseen molecules that do not exist in the other datasets. This reinforces the idea that existing difficulties with SMILES strings can be overcome by combining transformers with RL.

## Discussion

In this paper, we propose a solution for the de-novo drug design problem of generating novel molecules with desired properties. We introduce Taiga, a transformer-based architecture for the generation of molecules with desired properties. Taiga uses a two-stage approach by first learning a language modeling task and then optimizing for the desired properties using RL.

Our results demonstrate that the use of an attention mechanism enabled our method to overcome the problem of generating invalid SMILES strings. When compared to an RNN using the same RL technique, Taiga achieved similar or better results on all metrics across all of the examined datasets.

While all of the examined methods try to achieve the highest QED scores by directly optimizing for it on the generation task, Taiga outperformed state-of-the-art methods when generating molecules with the highest QED scores and obtained similar or better results in terms of validity, novelty, and diversity when generating arbitrary molecules.

When optimizing for biological properties like pIC50, Taiga reduced the concentration required by a factor of 3–5 for the same therapeutic effect (evaluated by an external property predictor), while maintaining similar scores on all other metrics, when using all datasets as baselines for training. When optimizing for anti-cancer activity, Taiga manages to achieve better anti-cancer activity while maintaining similar scores on all other metrics. Additionally, when examined by expert pharmacologists, several of the top molecules generated we evaluated as easily synthesizable and have a high chance of having anti-cancer properties, this emphasise the advantage of the RL stage, which allows us to optimize properties that are not derived from the model itself.

Our proposed method for molecule generation can enhance the drug development process by generating candidate molecules with improved therapeutic properties that are better than those of existing drugs on the market. The drug development process takes an average of 13 years, of which half are spent searching for lead molecules, and our proposed method can help reduce the time devoted to this task.

Future work can explore reward function that try to optimize several properties or doing constrained optimization from existing molecules.

## Methods

### Problem formulation

We define the molecule generation and optimization tasks similarly to the formulations used by the authors presenting GraphDF^19^. Given a set of molecules $\{m_i\}_{i=1}^M$ and a score function $R(m) \rightarrow \mathbb {R}$, the molecule generation task is defined as learning a generation model $p_\theta (\cdot )$, such that $p_\theta (m)$ is the probability of generating molecule *m*. The optimization task is defined as maximizing $\mathbb {E}_{M \sim p_\theta }[R(m)]$ with respect to *R* (in the context of molecules, *R* can be the IC50 of the molecule or or any property one might want to maximize).

### Overview

Figure 1 illustrates our proposed method. Taiga is based on a two-stage process in which we first train a transformer-based architecture on a general language modeling task by having the model predict the next token in the sequence of SMILES strings. Then we apply policy gradient RL (specifically, the REINFORCE algorithm) to achieve the desired molecular properties by learning a policy that maximizes the desired property as the reward in the RL stage. The main advantage of our proposed method is its utilization of a pretrained model capable of learning both the underlying rules of chemistry and the grammar of SMILES strings, which acts as an initial policy by training on the next-character prediction task. This improves the model when applying the REINFORCE algorithm.

### Language model

Similar to MolGPT^2^, we use a GPT-like decoder-based transformer model as an auto-regressive model for language modeling. The model consists of several decoder-only blocks stacked one after another. Each block uses the self-attention mechanism. This attention mechanism takes a set of keys, queries, and values (*q*, *k*, *v*) as inputs, applies a dot product operation between the queries and the keys, and then computes the attention weights for the values by using the softmax function on the result of the dot product. The attention mechanism is formulated as follows:

4 $$\begin{aligned} Attention(Q,K,V) = softmax(QK^T/\sqrt{d})V, \end{aligned}$$

In order to learn different representations, we use MultiHeadAttention, which allows us to attend information for different positions at the same time:

5 $$\begin{aligned} MultiHead(Q,K,V)= & {} Concat(head_1,\ldots ,head_n)W^o, \end{aligned}$$

6 $$\begin{aligned} head_i= & {} Attention(QW_i^Q, KW_i^K, VW_i^V), \end{aligned}$$

where $W_i^Q, W_i^K, W_i^V$ are the projection matrices of head i. To train a model that can carry out text generation tasks, we mask future tokens to prevent tokens from attending consecutive tokens when computing the self-attention mechanism.

We then define a transformer decoder block as follows:

7 $$\begin{aligned} \begin{aligned} z_l&= x_{l-1} + MHA(LayerNorm(x_{l-1})) \\ x_l&= z_l + MLP(LayerNorm(z_l)) \end{aligned} \end{aligned}$$

where $x_{l-1}$ is the input from the previous block, MLP is a multi-layer feed-forward network and MHA is the MultiHeadAttention defined previously. We can then stack as many layers of decoder blocks as we want to create out model.

### Reinforcement learning

In this subsection, we formulate the RL problem for molecule graph generation. We define the SMILES generation as a Markov decision process $M = (S, A, P, R, \gamma )$.

- **Observation Space:** A single state is represented as a vector *F*, we assume there is a finite set of character types that can be used to represent a SMILES string bounded by *n*, in which $F \in R^l$ where $f_i$ belongs to $\{0,\ldots ,n\}$. $S = \{s_i\}$ is the state space of all possible intermediate SMILES strings with length $t \le T$; *T* denotes the terminal state after the model generates a [EOS] token or reaches a maximal length; and $s_0$, which is the initial state, is an empty string.
- **Action Space:** $A = \{a_i\}$ is the set of actions the agent can take. In our case, all of the possible actions are the characters in the vocabulary you can append to the SMILES representation of the molecule, so we assume that $a_i$ belongs to $\{0,\ldots ,n\}$.
- **Transition Dynamics:** *P* is the transition dynamics that specify the probability of reaching a certain state given the current state and the chosen action, $p(s_{t+1} | s_t, a_t)$, since the state and action space consist of only characters; the transition dynamics are simply $p(s_{t+1} | s_t, a_t) = 1$, since appending a character is deterministic.
- **Reward Function:** *R* is the reward function for a given molecule. We define the reward as zero for all intermediate states, $R(s_t) = 0$. $R(s_T)=f(s_T)$ is a function applied to the molecule generated, and $\gamma$ is the discount factor.

### Policy gradients

We can now define the task of finding the set of parameters for our transformer-based network which maximizes the expected reward of our objective function $J(\theta )$:

8 $$\begin{aligned} \max _\theta J(\theta ) = \sum _{s \in S}d^\pi (s)V^\pi (s)=\sum _{|s|=T}d^\pi (s)V^\pi (s), \end{aligned}$$

where $d^\pi$ is the state distribution and $V^\pi$ is the value function. Since it is unreasonable to compute the sum of all terminal states, which are all of the states that end with the [EOS] token, due to the large number of terminal states, we sample them. Based on the rule of large numbers, we can approximate this sum. Then we determine the gradient of the expected value using policy $\pi _\theta (a|s)$.

9 $$\begin{aligned} \nabla _\theta J(\theta ) = \mathbb {E}_\pi [Q^\pi (s,a) \nabla _\theta \ln \pi _\theta (a|s)] = \mathbb {E}_\pi [G_t\nabla _\theta \ln \pi _\theta (a|s)] \end{aligned}$$

where $G_t$ is the return of the trajectory and is defined as:

10 $$\begin{aligned} G_t = R_{t+1}+\gamma R_{t+2}+\gamma ^2 R_{t+2}+\cdots +\gamma ^{T-1}R_T. \end{aligned}$$

## Supplementary Information

Supplementary Information.

## Data Availability

The ZINC, Moses and GDB13 datasets used in this study are available online. The code is available at https://github.com/eyalmazuz/MolGen.
