# Supplementary material for: Molecule generation using transformers and policy gradient reinforcement learning
Source: Sci Rep. 2023 May 31;13:8799. doi: 10.1038/s41598-023-35648-w (PMC10232454; doi:10.1038/s41598-023-35648-w)
Supplement: Supplementary file 1 — Supplementary Information. [file 41598_2023_35648_MOESM1_ESM.pdf]

Molecule Generation Using Transformers and Policy Gradient Reinforcement Learning- Supplementary Information

Eyal Mazuz, Guy Shtar, Bracha Shapira, Lior Rokach

April 15, 2023

Table S1: Performance comparison on the property generation task.

Dataset	Model	QED 1st(↑)	QED 2nd(↑)	QED 3rd(↑)	SAS 1st(↓)	SAS 2nd(↓)	SAS 3rd(↓)
GDB13	GCPN	-	-	-	-	-	-
	JTVAE	0.786	0.786	0.775	4.35	4.57	4.15
	MolGPT	0.860	0.840	0.830	3.54	3.68	3.65
	MolGAN	0.732	0.732	0.732	3.68	**3.25**	**3.25**
	GraphDF	0.739	0.726	0.723	4.67	6.46	5.66
	LSTM-PG	0.870	0.842	0.826	**3.94**	4.11	4.00
	Taiga	**0.914**	**0.891**	**0.891**	4.17	3.91	4.25
Moses	GCPN	0.943	0.941	0.940	1.95	4.04	3.06
	JTVAE	0.881	0.872	0.872	3.05	4.16	4.00
	MolGPT	**0.948**	**0.948**	0.947	2.77	2.51	3.14
	MolGAN	-	-	-	-	-	-
	GraphDF	0.804	0.802	0.798	4.35	5.12	3.83
	LSTM-PG	0.948	0.947	0.947	**1.83**	**2.44**	**1.71**
	Taiga	**0.948**	**0.948**	**0.948**	2.37	2.62	2.90
Zinc	GCPN	0.931	0.929	0.929	4.86	**2.29**	4.71
	JTVAE	0.871	0.871	0.871	3.45	5.35	5.20
	MolGPT	0.947	0.946	0.946	4.68	4.36	3.88
	MolGAN	0.339	0.339	0.339	5.23	5.23	5.23
	GraphDF	0.804	0.783	0.769	4.96	4.40	4.61
	LSTM-PG	0.810	0.790	0.770	**2.07**	3.65	2.27
	Taiga	**0.948**	**0.947**	**0.947**	2.27	3.26	**1.77**
-	MolDQN	0.947	0.947	0.946	5.08	5.08	5.61

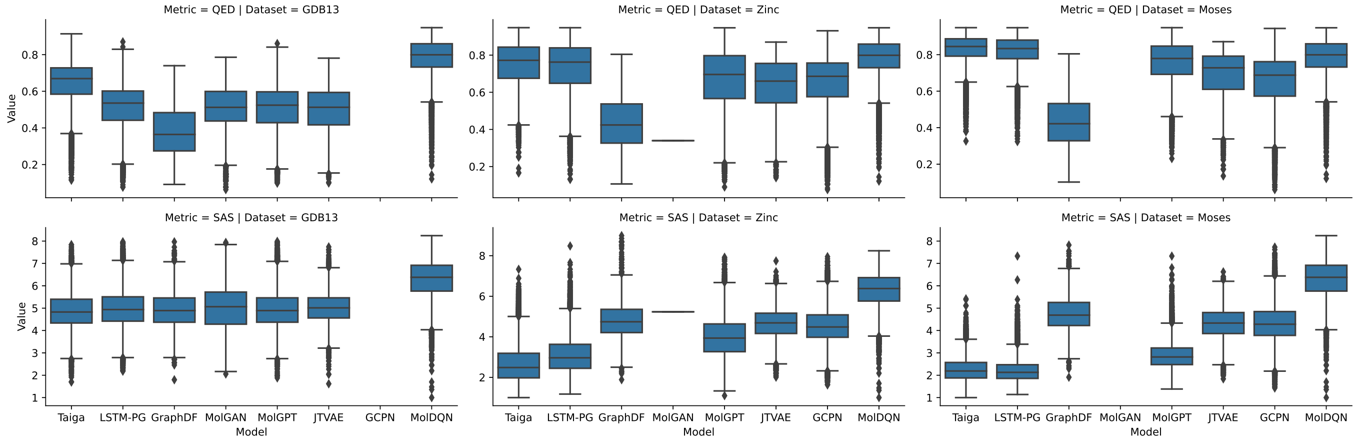

Figure S1: Performance comparison on the property generation task; the mean score and standard deviation of the molecules is presented. As to not create a separate sub-figure for MolDQN we added it to each plot respectively.

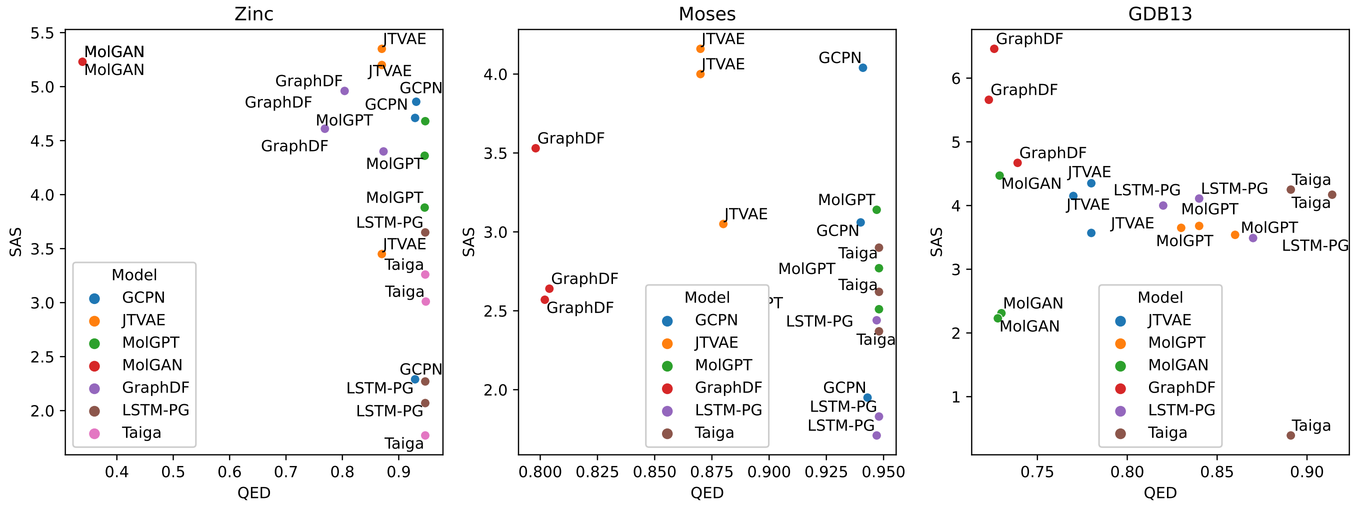

Figure S2: Top K molecule performance of each model. We plotted the top 3 molecules the model generated in terms of QED and calculated their respective SAS score, each subplot is a different dataset.

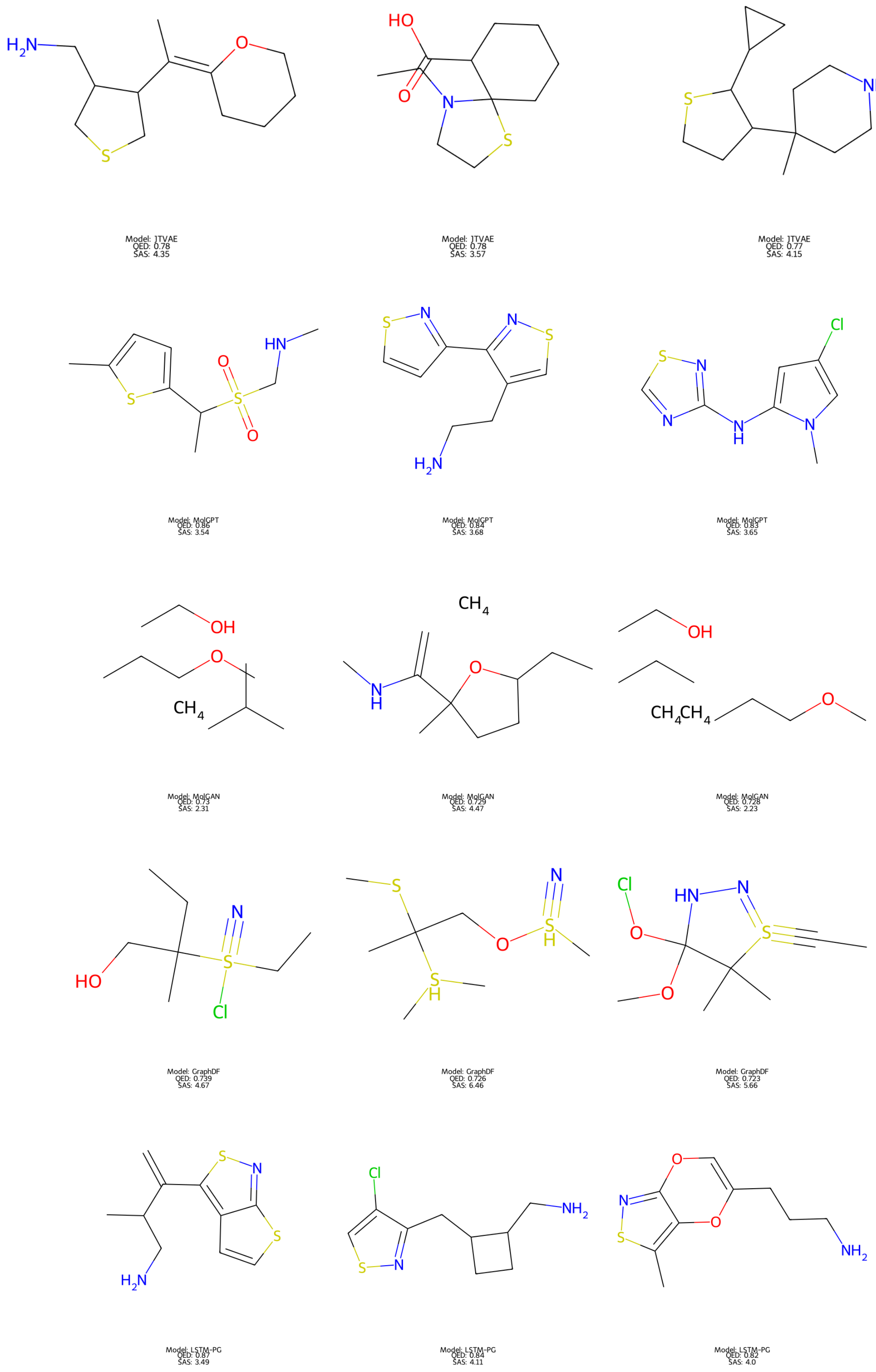

Figure S3: Visualization of the top 3 molecules each model generated for each of our 3 datasets

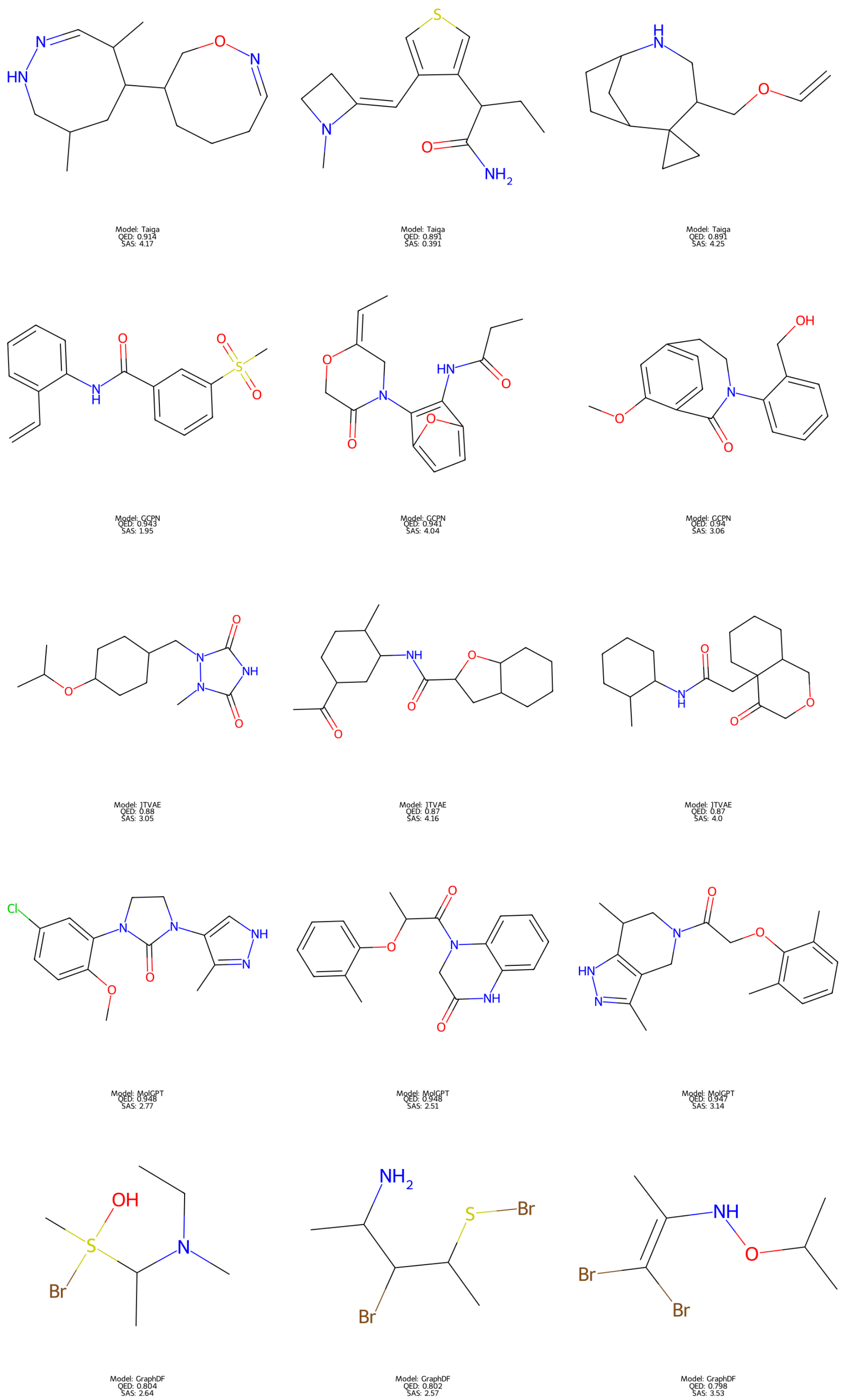

Figure S4: Visualization of the top 3 molecules each model generated for each of our 3 datasets

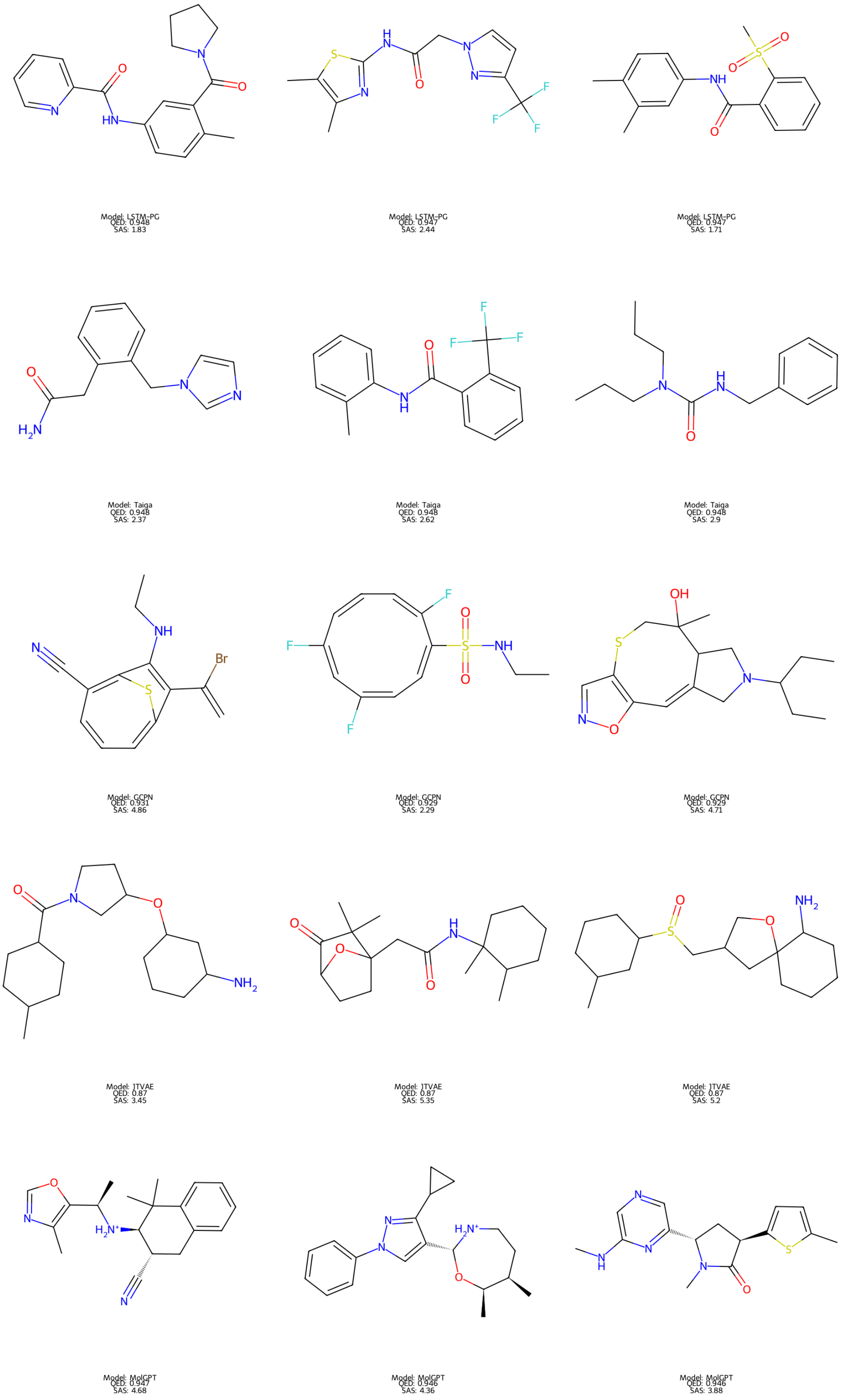

Figure S5: Visualization of the top 3 molecules each model generated for each of our 3 datasets

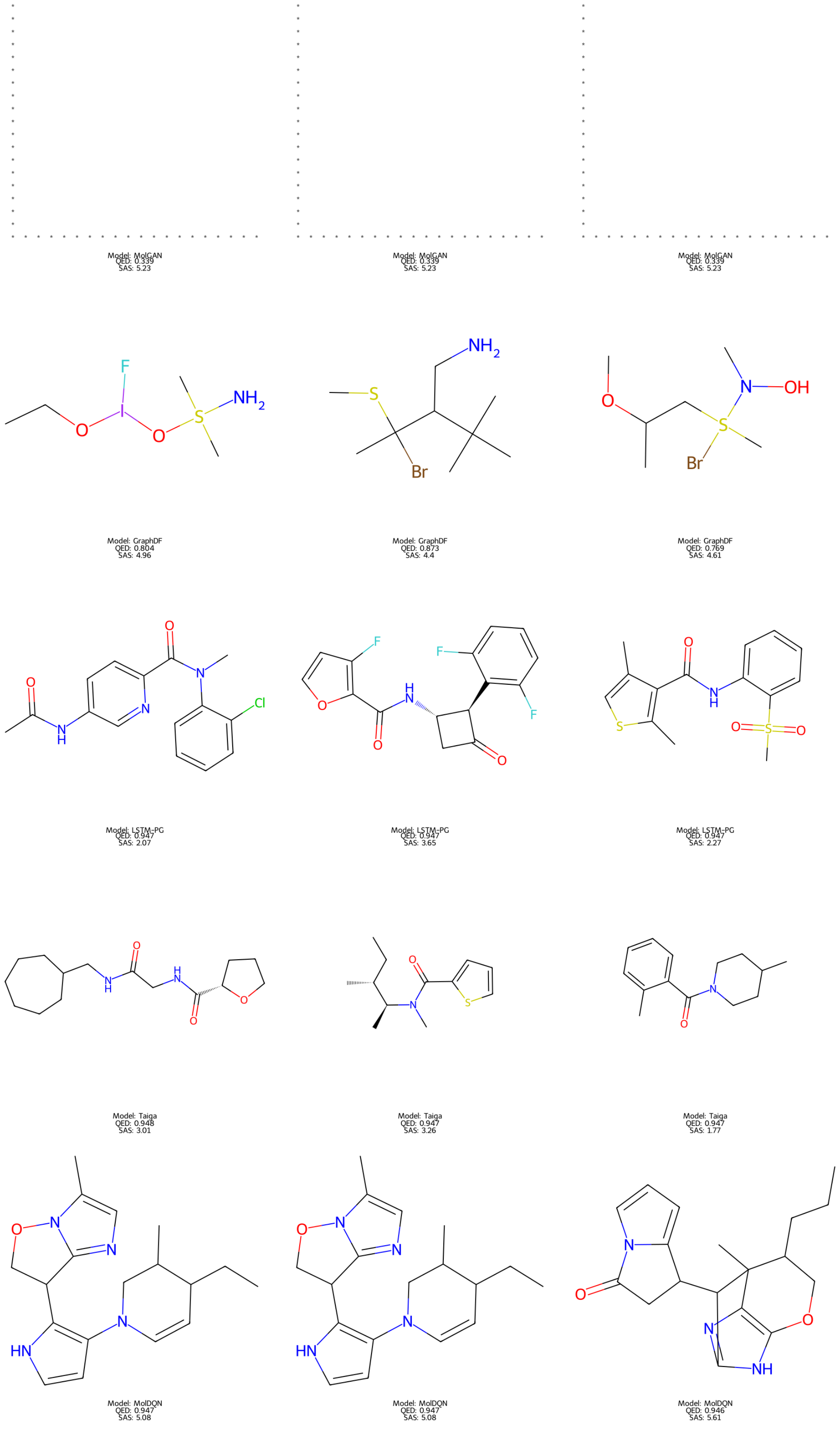

Figure S6: Visualization of the top 3 molecules each model generated for each of our 3 datasets
